# Feeling Stress: The Mechanics of Cancer Progression and Aggression

**DOI:** 10.3389/fcell.2018.00017

**Published:** 2018-02-28

**Authors:** Josette M. Northcott, Ivory S. Dean, Janna K. Mouw, Valerie M. Weaver

**Affiliations:** ^1^Department of Surgery, Center for Bioengineering and Tissue Regeneration, University of California, San Francisco, San Francisco, CA, United States; ^2^Department of Radiation Oncology, University of California, San Francisco, San Francisco, CA, United States; ^3^Department of Bioengineering and Therapeutic Sciences, University of California, San Francisco, San Francisco, CA, United States; ^4^Eli and Edythe Broad Center of Regeneration Medicine and Stem Cell Research, University of California, San Francisco, San Francisco, CA, United States; ^5^UCSF Helen Diller Comprehensive Cancer Center, University of California, San Francisco, San Francisco, CA, United States

**Keywords:** cancer progression, cell contractility, mechanical stresses, tissue tension, solid stress, ECM stiffness, therapeutic targets

## Abstract

The tumor microenvironment is a dynamic landscape in which the physical and mechanical properties evolve dramatically throughout cancer progression. These changes are driven by enhanced tumor cell contractility and expansion of the growing tumor mass, as well as through alterations to the material properties of the surrounding extracellular matrix (ECM). Consequently, tumor cells are exposed to a number of different mechanical inputs including cell–cell and cell-ECM tension, compression stress, interstitial fluid pressure and shear stress. Oncogenes engage signaling pathways that are activated in response to mechanical stress, thereby reworking the cell's intrinsic response to exogenous mechanical stimuli, enhancing intracellular tension via elevated actomyosin contraction, and influencing ECM stiffness and tissue morphology. In addition to altering their intracellular tension and remodeling the microenvironment, cells actively respond to these mechanical perturbations phenotypically through modification of gene expression. Herein, we present a description of the physical changes that promote tumor progression and aggression, discuss their interrelationship and highlight emerging therapeutic strategies to alleviate the mechanical stresses driving cancer to malignancy.

## Introduction

The tumor microenvironment is a dynamic landscape composed of cancer cells surrounded by the extracellular matrix (ECM) and a host of stromal cells, including fibroblasts, immune cells, blood and lymphatic vascular cells, and other tissue-specific cells (e.g., adipocytes). From transformation of the normal tissue, on through progression of the primary tumor to invasion, dissemination, and metastasis, the physical context of the tumor changes dramatically (Kumar and Weaver, 2009). These changes to the tumor microenvironment are driven by enhanced tumor cell contractility, expansion of the growing tumor mass, and alterations to the material properties of the surrounding ECM. Indeed, the physical properties of a tissue, such as ECM stiffness and architecture, can have a profound influence on cellular behavior and ultimately, tissue organization and function.

**Figure 1:**
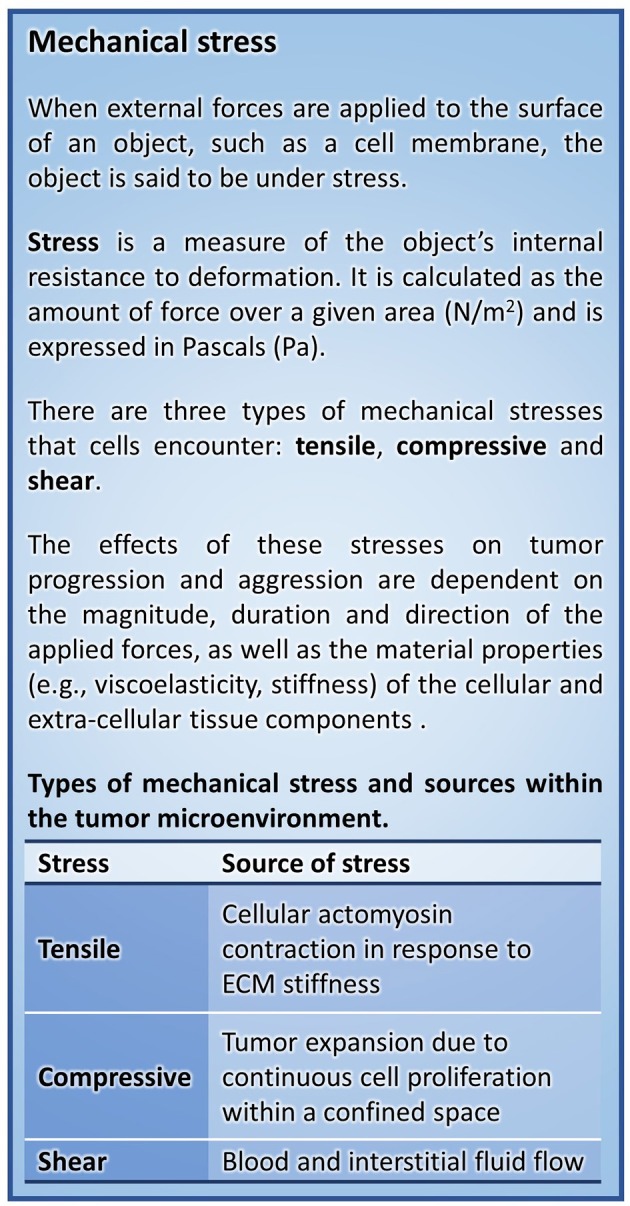
Mechanical stress.

**Figure 2:**
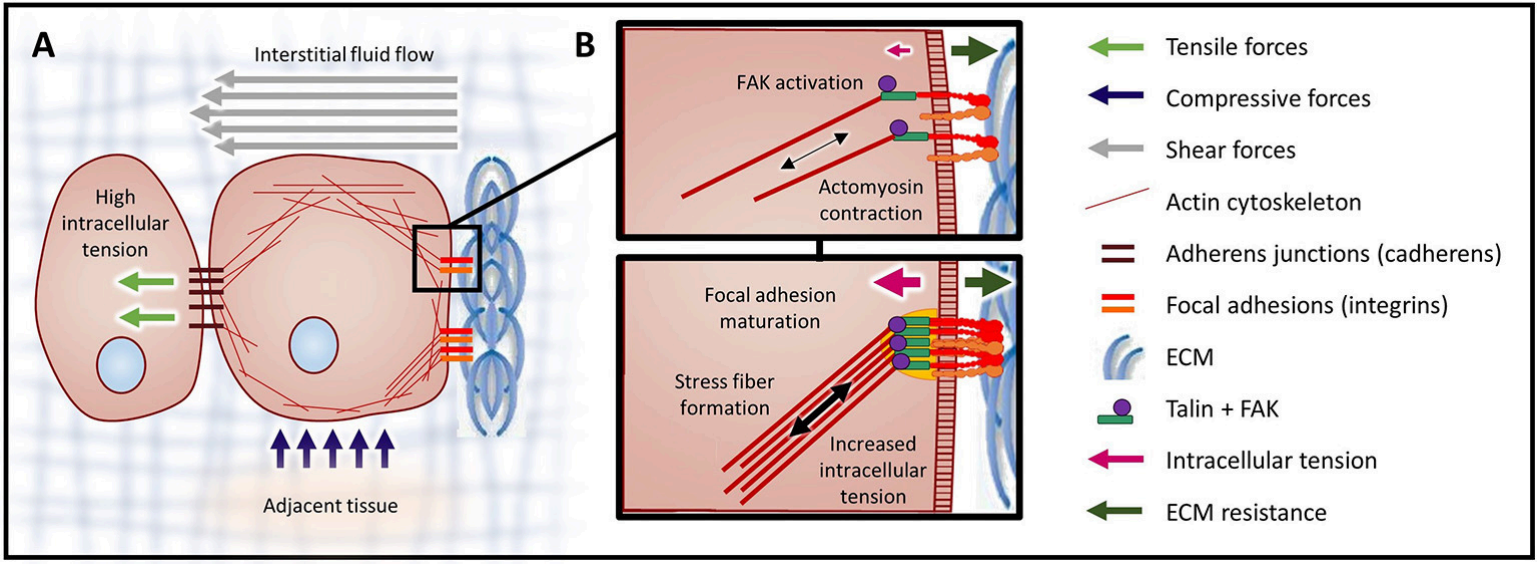
Mechanical forces and cellular contractility. **(A)** Forces exerted onto a cell can cause three types of mechanical stress: tensile (stretch), compressive, and shear. Forces that induce tensile and compressive stress are applied perpendicular to the cell surface, while forces causing shear stress are exerted parallel to the cell surface. **(B)** Integrin clusters activated through ECM binding are bound by talin, initiating actin polymerization and producing intracellular tension. In response to high tensile force (a consequence of elevated ECM rigidity), FAK is activated (top). Activation of FAK leads to the recruitment of additional linker proteins, focal adhesion maturation, and increased actomyosin contraction via the assembled actin stress fibers (bottom).

Cancer progression is associated with changes to both the cellular responses to chemical and mechanical signals as well as to the material properties of the ECM components of the transformed tissue (reviewed in Butcher et al., 2009; Kumar and Weaver, 2009). Cells are exposed to a number of different mechanical stresses (Figures 1, 2A) that activate downstream signaling pathways through a process termed mechanotransduction. Importantly, oncogenes engage many of the same signaling pathways that are activated in response to mechanical stress (reviewed in Yu et al., 2011), thereby reworking the cell's intrinsic response to exogenous mechanical stimuli and enhancing cytoskeletal contractility. Active tumor cell engagement with this mechanically-evolving microenvironment results in changes to cytoskeletal structure, cellular shape, differentiation, survival/death, proliferation, adhesion, and migration which can drive tumor progression and aggression (Butcher et al., 2009; Yu et al., 2011). Herein we present a description of the mechanical stresses that promote tumor progression and aggression, discuss their interrelationship, and highlight emerging therapeutic strategies to alleviate the mechanical stresses driving cancer to malignancy.

## Tumor cell contractility

Cells respond to mechanical stresses by altering their intracellular tension (Figure 2). This response is achieved through coordinated cytoskeletal rearrangement and actomyosin contraction. Mechanotransduction is the process through which cells sense and respond to mechanical signals (e.g., ECM rigidity, compression, tension) by translating these mechanical stimuli into biochemical signals. Cellular interpretation of these biochemical signals influences cell morphology, behavior and function.

### Mechanotransduction and actomyosin contractility

Mechanotransduction is essential for a number of normal biological processes, such as hearing (reviewed in Schwander et al., 2010), and pathologies, such as cancer. Focal adhesion and adherens junction protein complexes mediate the bi-directional physical communication between cell-ECM and neighboring cells, respectively. In this way, ECM rigidity and intercellular dynamics modify intracellular tension, which feeds back to influence tissue stiffness and morphology. Application of forces to the cell membrane can also lead to opening of mechanically-activated ion channels (e.g., Piezo1 and Piezo2) and actomyosin contractility (Maroto et al., 2012, reviewed in Honore et al., 2015).

Cells interact with the ECM via integrin receptors and, in response to mechanical signals from the ECM, integrins become activated and oligomerized (Butcher et al., 2009). Subsequently, the adhesion plaque protein talin undergoes a conformational change, which fosters intermolecular interactions. Once the integrin-adhesion plaque association has been activated, the focal complexes begin to assemble and eventually mature into focal adhesions (Butcher et al., 2009). Focal adhesions are composed of multiple mechanosensors (e.g., talin, vinculin), signaling molecules [e.g., focal adhesion kinase (FAK), SRC, phosphatidylinositol-3 kinase (PI3K)], adapter proteins (e.g., paxillin) and actin linker proteins (e.g., filamin, alpha-actinin) which physically connect the integrins to the cytoskeleton (reviewed in Wozniak et al., 2004). On stiff substrates, physical resistance to cellular tension enhances talin stabilization through vinculin binding and also potentiates FAK activation (Figure 2B). Focal adhesion formation results in Rho/Rho kinase (ROCK) activation and actomyosin contraction. Reinforcement / maturation of the focal adhesion complex further increases contraction via the assembly of actin stress fibers (Figure 2B). In addition, via interplay with growth factor receptors (GFRs) and G-protein coupled receptors (GPCRs), focal adhesion formation also activates signaling downstream of both FAK and SRC (e.g., MAPK) (Butcher et al., 2009; Yu et al., 2011).

The magnitude of these transmitted mechanical signals is regulated by the stabilization of the focal adhesions (greater number or size), which leads to augmented actomyosin contractility and traction force generation. Focal adhesion formation and FAK activation synchronizes the immediate response (traction force generation) and the sustained response (altered gene expression) through coordinated actomyosin contraction and signaling pathway activation, respectively. Increased cellular tension, integrin signaling and crosstalk with GFR and GPCR signaling pathways, ultimately lead to enhanced tumor cell growth, survival, and invasion (Provenzano and Keely, 2011).

Similar to the focal adhesions, the adherens junction complexes that communicate intercellular tension are composed of receptors (i.e., cadherins), mechanosensors (i.e., catenins), linker proteins, and signaling molecules (i.e., SRC). Adherens junctions and focal adhesion complexes crosstalk though shared protein components and physical interconnection via the actin cytoskeleton (Oldenburg et al., 2015, reviewed in Mui et al., 2016). Indeed, actomyosin contractility-induced cell tension can promote tissue stiffness and β-catenin mediated interfollicular epidermal hyperplasia and tumor growth (Samuel et al., 2011).

In addition, mechanotransduction can occur through mechanically-activated ion channels (e.g., Piezo1), which respond to external stimuli either directly or indirectly. Mechanically-activated ion channels, which are regulated by GTPases, were among the first mechanosensor proteins described. Like other chemical mechanotransduction pathways, signals are amplified within the cell and crosstalk with other signaling pathways. These proteins regulate many cellular processes, such as cell polarity (Huang et al., 2015) and proliferation (Basson et al., 2015) that are perturbed in cancer. Recently, Gudipaty and colleagues demonstrated that stretch-induced epithelial cell division and extrusion require activation of Piezo1 (Gudipaty et al., 2017). Mechanically-activated ion channels, including Piezo1, have been shown to be dysregulated in both solid (Maroto et al., 2012; McHugh et al., 2012; Yang et al., 2014; Fels et al., 2016) and non-solid tumors (Morachevskaya et al., 2007; Nam et al., 2007; Pottosin et al., 2015), implicating this mode of mechanotransduction as a potential driver of tumor progression.

### Reciprocity of intracellular tension and gene expression

Sustained cellular responses to mechanical stimuli depend ultimately upon altering gene expression. For example, expression of integrins, matrix metalloproteases (MMP), and ECM proteins is increased in response to stiff substrates (Delcommenne and Streuli, 1995; Nukuda et al., 2015). These gene expression changes enable cells to modify the compliance of their microenvironment by remodeling the ECM via increased tension and altered matrix composition and arrangement. Force-induced gene expression changes can also affect the mechanical properties of the tumor cell themselves by altering the expression of cytoskeletal proteins (McGrail et al., 2015).

The magnitude of intracellular tension generated by actomyosin contractility also depends on the cytoskeletal composition (type and organization of actin, intermediate filament and microtubule proteins), which reflects the cell type and phenotypic state of the cell (Butcher et al., 2009; McGrail et al., 2015). The cytoskeletal architecture, intracellular tension and force-generating capabilities of transformed cells are notably changed from the normal epithelium. Intermediate filaments, which provide mechanical support to the cell, differ in their expression profile between normal and transformed epithelial cells (Kumar and Weaver, 2009). Furthermore, epithelial-to-mesenchymal transformation (EMT), a proposed feature of highly metastatic tumor cells, is characterized by a switch from keratin to vimentin intermediate filament expression (reviewed in Kumar and Weaver, 2009; Wirtz et al., 2011). Changes to the cellular cytoskeleton, such as during transformation or EMT, drastically alter the shape and the amount of tension exerted on neighboring cells and the ECM. EMT results in cells with increased migration, invasion and dissemination potential (Yu et al., 2011).

In addition to mechanical signals being transduced to the nucleus through chemical mechanotransduction pathways (mechanosignaling), it is possible that physical signals may be directly transduced to the nucleus through physical anchoring of the cytoskeletal networks with the nuclear lamina via the linker of nucleoskeleton and cytoskeleton (LINC) complex (Crisp et al., 2006; Wirtz et al., 2011). This paradigm maintains that as actin is tethered to both the cell membrane as well as the nuclear membrane, tensional forces can be transmitted to the nucleus (via the LINC complex), neighboring cells (via adherens junctions) and the ECM (via focal adhesions). Interestingly, it was shown in isolated nuclei that the nucleus also responds to mechanical tension by inducing nuclear stiffening (Guilluy et al., 2014); however, what role this plays in the regulation of gene expression is currently unknown.

### Oncogene and growth factor modulation of cell contractility

Mechanotransduction pathways crosstalk with other chemical signal transduction cascades (e.g., GFR and GPCR signaling pathways) to influence transient responses and persistent cellular behaviors though modulation of gene expression (Butcher et al., 2009; Yu et al., 2011). For instance, stimulation of the thrombin GPCR Par1 causes RhoA/Rap1 mediated activation of integrin signaling, phosphorylation of FAK and ERK1/2, and increased glioblastoma cell proliferation (Sayyah et al., 2014). In invasive melanoma cells, cytokine signaling-induced activation of the JAK1/STAT3 pathway leads to increased ROCK-dependent actomyosin contractility, that feeds back to reinforce STAT3 signaling and promote ECM remodeling by tumor-associated stromal fibroblasts (Sanz-Moreno et al., 2011). Thus, mechanical forces collaborate with biochemical signals to modulate cell, and ultimately, tissue behavior.

Following transformation, a tumor cell's intrinsic response to tension is often altered by oncogene engagement with mechanotransduction components. For example, EGFR-transformed breast epithelial cells have elevated ERK-Rho activity and increased myosin-mediated cell contractility (Paszek et al., 2005). Furthermore, HRAS activation of ROCK drives the progression of squamous cell carcinoma by inducing actomyosin contractility, tissue stiffness (through collagen deposition) and cell proliferation (Samuel et al., 2011). Moreover, the influences of oncogene expression and growth factor/cytokine stimulation can converge onto mechanotransduction pathways to alter tumor progression. In a KRAS-driven murine pancreatic ductal carcinoma (PDAC) model, loss of TGF-beta signaling leads to activation of GPCR-mediated JAK/STAT3 pathway signaling and stimulation of ROCK1, that increases tumor cell contractility, induces ECM remodeling and localized stiffening, and promotes focal adhesion maturation to promote tumor progression and aggression (Laklai et al., 2016). These studies highlight the common link and between increased actomyosin contractility of cells within the tumor microenvironment, augmented ECM stiffness and cancer progression across a variety of solid tumors from various organs (i.e., breast, skin, and pancreas).

### Tension-regulated cellular processes drive tumor progression

**Figure 3:**
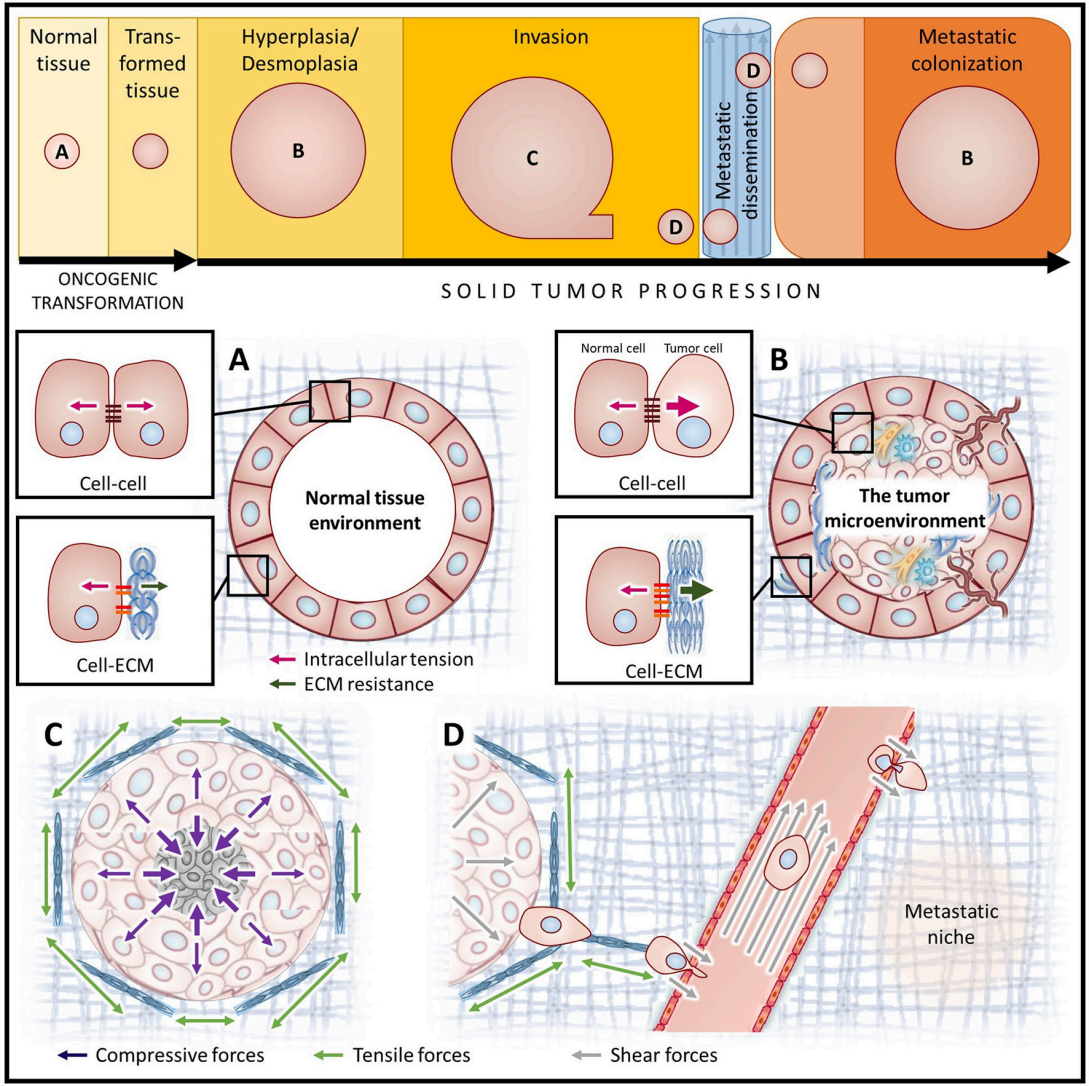
Diverse mechanical stimuli act on tumor cells throughout cancer progression. Simplified depiction of oncogenic transformation and solid tumor progression for cancers of epithelial origin (top). Letters indicate the stages of cancer progression focused on in panels **(A–D)**. **(A)** In homeostatic tissues, the forces between cells and the ECM are balanced. **(B)** The tumor microenvironment is composed of cancer cells with augmented contractility (increased intracellular tension), surrounded by a progressively stiffening ECM (increased ECM resistance), and a host of stromal cell types including fibroblasts, immune cells and vascular cell types. **(C)** Tumor expansion confined by the surrounding stroma compresses both the tumor and the adjacent stromal tissue, causing increased interstitial pressure. Augmented ECM rigidity increases stromal resistance to compression and exacerbates solid stress. **(D)** A high interstitial fluid pressure gradient elicits fluid flow from the tumor core to the periphery, promoting metastatic dissemination. Following escape from the primary tumor, cancer cells migrate along tension-oriented collagen fibers toward the vasculature. Tumor cells are exposed to high shear stresses as they intravasate/extravasate between endothelial cells and travel through the circulation *en route* to future secondary tumor sites.

Increased intracellular tension through actomyosin contractility is sufficient to induce cell proliferation, disrupt cell–cell adherens junctions and effect ECM remodeling, all of which can promote loss of tissue polarity, ECM reorganization/stiffening and tumor cell invasion (Butcher et al., 2009; Yu et al., 2011) (Figure 3). Transformed cells exert higher traction forces than normal cells when embedded within a compliant matrix resembling physiological stiffness conditions (Wang et al., 2000; Paszek et al., 2005; Butcher et al., 2009). The ability of a cell to invade depends upon both the magnitude and direction of traction forces (Koch et al., 2012).

In addition to a cell's ability to generate traction, metastasis necessitates that cells are able to squeeze through small openings in the ECM and between cells of the endothelium (Wirtz et al., 2011). Cell compliance was demonstrated to be tuned by the extracellular context, as tumor cells stiffen as they invade into 3D collagen gels due to increased actomyosin contractility (Staunton et al., 2016). Thus, it is not surprising that tumor cells may become more compliant than their normal counterparts and that the extent of cellular compliance correlates with metastatic capability (Guck et al., 2005). Interestingly, higher target cell membrane tension enhances perforin-mediated killing by cytotoxic CD8+ T cells (Basu et al., 2016), suggesting that increased compliance of metastatic tumor cells could potentially enable them to evade immune destruction.

While the current perspective is that cells need to be softer to enable migration under spatial constraint, a recent study demonstrated that the nucleus is the greatest impediment to confined migration, not cortical tension (Mekhdjian et al., 2017). This finding suggests that the nucleus, not the cortex, of metastatic tumor cells is softer and that this deformability, together with the ability to exert higher traction force at the integrin adhesions, permits metastatic cells to navigate rapidly through confined stiff spaces. Thus, the ability of cells to migrate through a dense ECM depends on adhesiveness, nuclear volume, contractility, and to a lesser extent cortical cell stiffness (Lautscham et al., 2015). Indeed, the majority of total cell stiffness comes from the nucleus, which is the largest organelle and almost an order of magnitude stiffer than the cytoplasm (Dahl et al., 2004; Tseng et al., 2004). As cells migrate through dense matrices, the nucleus must deform, which can cause nuclear rupture and DNA damage to occur (Denais et al., 2016; Raab et al., 2016). Decreasing nuclear stiffness, through knockdown of lamin A expression, increases cell motility and ability to migrate through dense matrices but impairs the survival of tumor cells exposed to shear stress (Davidson et al., 2014; Mitchell et al., 2015). Thus, greater nuclear compliance coupled with elevated contractile forces enables cells to pull themselves through tight spaces with less risk of nuclear rupture.

Another form of cellular deformation involved in cell migration that depends on actin cytoskeleton rearrangement is invadopodia formation. Invadopodia are linked to tumor cell invasion and metastasis, and augmenting intracellular tension (using the PP1/2 inhibitor calyculin A) increases invadopodia formation, protease secretion, ECM degradation and an invasive cellular phenotype (Aung et al., 2014; Jerrell and Parekh, 2014). Thus cellular tension generation plays important roles in tumor metastasis.

## Microenvironmental stresses

### Solid stress

Unchecked proliferation of cancer cells results in rapid expansion of the tumor mass, compression of the tumor interior and distention of the surrounding stromal tissue. The forces exerted by the expanding tumor mass and the resistance to deformation of the surrounding stromal tissue make up what is collectively known as solid stress (reviewed in Jain et al., 2014; Figure 3). Recently, several new methods have been developed to measure solid stress in tumors which have demonstrated that the tumor type, tumor size and the properties of the surrounding tissue all influence tumor solid stress (Nia et al., 2016).

Forces and strains propagated outward from the tumor, toward the surrounding stromal tissue, can result in increased ECM tension and remodeling, as well as disruption of tissue structure surrounding the tumor mass (Jain et al., 2014). Elevated ECM tension in these adjacent tissues may be exacerbated by crowding from tumor-associated myofibroblast proliferation and immune cell infiltration/expansion during the desmoplastic and pro-inflammatory stromal responses. Furthermore, changes to the material properties of the ECM (i.e., stiffening due to deposition/remodeling) can also contribute to the growth and solid stress of the tumor. Collagen fibers stiffen under tension causing resistance to further stretching, while hyaluronan can trap interstitial fluid and swell due to hydration, providing resistance to compression and increased intratumoral solid stress (Jain et al., 2014). In order for a tumor to continue to increase in size, it must displace or degrade the surrounding non-malignant tissue. Computational modeling has found that the stiffness of a solid tumor must exceed 1.5 times that of the surrounding tissue in order to continue to expand (Voutouri et al., 2014).

Conversely, externally-applied forces resulting in compression or confinement of the tumor volume may reduce cancer cell proliferation, induce apoptosis/necrosis, augment ECM deposition/organization, and enhance the invasive and metastatic potential of tumor cells (Yu et al., 2011). Indeed, externally-applied compressive stress is sufficient to reduce the volume and proliferative rate of cells in the core of multicellular structures grown in 3D matrices (Helmlinger et al., 1997; Delarue et al., 2014; Mascheroni et al., 2016). Low-proliferating cell populations contribute to treatment resistance and compression of tumor cells themselves may compromise the efficacy of chemotherapeutic agents (Mascheroni et al., 2017). *In vivo*, 1 month of induced compression stress (at levels comparable to those measured in growing tumors), leads to translocation of β-catenin from the adherens junctions to the nucleus, activation of β-catenin target genes, and increased colon crypt size (due to hyperplasia) (Fernández-Sánchez et al., 2015), implicating compression stress as an inducer of tumorigenesis.

Moreover, solid stress compresses blood vessels in the tumor interior, which impairs the oxygen and nutrient supply to the tumor and temporarily impedes cancer progression (Padera et al., 2004). Sustained compression of the vasculature within the tumor results in poor tissue perfusion, hypoxia and the development of a necrotic core (Stylianopoulos et al., 2013). Hypoxia in the tumor core drives metastasis directly by increasing EMT and stem-like features in cancer cells (reviewed in Muz et al., 2015), as well as indirectly, through recruitment of pro-tumor macrophages (reviewed in Condeelis and Pollard, 2006). Together, this increases the invasive and metastatic potential of tumor cells.

Solid stresses at the periphery of the tumor are sufficient to cause compression of blood vessels surrounding the tumor and consequently, vessels in the surrounding normal tissue are deformed to elliptical shapes (Stylianopoulos et al., 2013). Constriction of the lymphatic vasculature within the stroma reduces drainage of extravasated fluid (Jain et al., 2014). Thus, solid stress may promote elevated interstitial fluid pressure as a consequence of reduced interstitial space and compromised collection of fluid due to blocked vessels. In the context of a confined tumor, such as glioblastoma multiforme (GBM), this stress may be intensified by confinement of the brain by the skull. Together, reduced blood flow and increased interstitial pressure hinders efficient drug delivery of chemotherapeutics to the tumor and exacerbates hypoxia (Zhang et al., 2014), which reduces the efficacy of radiation treatment (Mpekris et al., 2015).

Mathematical modeling predicts that changes in the collective tension (a product of cell–cell adhesion) at the tumor-stoma interface can affect the shape of the tumor and this loss of tension can promote collective cell migration and metastasis (Katira et al., 2013). Thus, rate and direction of tumor cell migration may also be affected by the solid stress dynamics.

### Shear stress

The metastatic process subjects tumor cells to a variety of additional microenvironments and forces. As tumor cells escape the primary tumor and transit through the circulation, they are exposed to a number of different solid and fluid forces, many of which elicit shear stress (Figure 3). Hemodynamic shear stress, which is caused by the movement of blood along the cell surface, is influenced by both the fluid viscosity and fluid flow velocities (Wirtz et al., 2011). Shear stress can also be caused by solid forces from endothelial cell contact as tumor cells intravasate and extravasate from the vasculature.

For metastasis to occur, tumor cells must survive transit within the circulation, and not surprisingly, tumor cells have been shown to be more resistant to shear stress than normal cells (Mitchell et al., 2015). Survival is dependent on the time spent in circulation and the magnitude of shear stress tumor cells experience (Fan et al., 2016). Physiological resting levels of shear stress [~5–30 dynes/cm^2^ (0.5–3 Pa)] inhibit proliferation and stimulate migration and adhesion of tumor cells (Avvisato et al., 2007; Mitchell and King, 2013; Ma et al., 2017; Xiong et al., 2017), while levels of shear stress similar to exercise conditions [60 dynes/cm^2^ (6 Pa)] caused tumor cell death (Regmi et al., 2017). Thus, the ability of a tumor cell to withstand the various dynamic mechanical stresses it will encounter, as it leaves the primary tumor and establishes a secondary tumor site, will require continuous cellular adaptation.

Metastatic sites are not random. Primary tumors from various organs show a preference for colonization of different secondary organs, likely due to a combination of blood flow pattern (mechanical hypothesis) and favorability of the microenvironment (seed and soil hypothesis) (Wirtz et al., 2011). Circulating tumor cell adhesion to the vessel wall is required for extravasation. High shear stress increases the frequency of tumor-endothelial cell contact, but impedes the ability of these cells to form stable cell–cell adhesions (Wirtz et al., 2011). High shear forces require tumor cells to make stronger adhesions in order to extravasate. Thus, often tumor cells extravasate at branch points or after being trapped in small capillaries (Wirtz et al., 2011).

Similar to how cells undergo drastic rearrangement of their cytoskeleton and deformation to squeeze themselves through dense ECM matrices (Wirtz et al., 2011), actomyosin contraction induced cellular tension likely plays a vital role in tumor cell resistance to shear forces, enabling survival in circulation and during extravasation between endothelial cells. Importantly, hemodynamic forces also affect the phenotype and function of endothelial cells composing the vasculature and thus impact cancer progression via this axis; however, this discussion is outside the scope of this review.

### Interstitial fluid pressure

In normal tissue, the interstitial fluid pressure is a physiological hydrostatic pressure composed of the pressure from both the free fluid and the fluid immobilized by hyaluronan. However, the interstitial fluid pressure within the tumor microenvironment drastically increases as a consequence of tumor growth, augmented vascular permeability (leakiness) and impaired lymphatic drainage (Stylianopoulos et al., 2013; Jain et al., 2014).

Tumour blood vessels are often tortuous with irregular branching morphologies and leaky due to a lack of pericyte coverage (reviewed in Carmeliet and Jain, 2011). These vascular abnormalities lead to inadequate blood flow rates, reduced oxygen tension (hypoxia) and increased interstitial fluid pressure (Carmeliet and Jain, 2011; Jain et al., 2014). Hyaluronan swelling (due to increasing fluid retention), is another major contributor to interstitial fluid pressure that can also intensify solid stress by providing compressive resistance to the ECM (Jain et al., 2014). Increasing solid stresses can lead to lymphatic vessel crushing and impaired fluid drainage (Padera et al., 2004). Together, these factors increase the interstitial fluid pressure within the tumor and contribute to a difference in fluid pressures between the tumor and the surrounding tissue (Jain et al., 2014).

The difference between the elevated interstitial fluid pressure within the tumor and interstitial pressure of the adjacent peritumoral tissue creates a fluid pressure gradient that causes the outward flow of fluid from the tumor into the surrounding stroma and may facilitate tumor cell escape from the primary tumor (Figure 3; Jain et al., 2007). Furthermore, while solid stresses impede blood flow and thereby inhibit perfusion, high interstitial fluid pressure limits the penetration and dissemination of therapeutic agents in solid tumors, impairing treatment efficacy (Boucher and Jain, 1992; Jain et al., 2007, 2014).

## ECM material properties

Cancer progression is associated with changes to tissue structure and mechanical properties of the ECM (Figure 3). Increased ECM rigidity (decreased compliance) correlates with cancer progression and is sufficient to perturb normal tissue morphology (Paszek et al., 2005).

ECM remodeling in the tumor involves ongoing production of matrix proteins, their assembly and crosslinking, as well as their turnover by MMPs. This remodeling contributes to ECM stiffening, which occurs primarily through increased collagen deposition (i.e., increased protein concentration), augmented collagen crosslinking (e.g., higher lysyl oxidase (LOX) enzyme expression), and via parallel reorientation of the collagen fibers (Butcher et al., 2009; Kumar and Weaver, 2009; Yu et al., 2011). Importantly, increased collagen abundance and reorganization into thick, linearly oriented fibers correlates with tumor progression and clinical outcome (Acerbi et al., 2015). High ECM stiffness may also predispose individuals to develop certain cancers. Normal breast tissue clinically determined to have high mammographic density (MD) contains stiffer ECM, thicker collagen fibers and more linearized collagen than low MD breast tissue (Acerbi et al., 2015), and was shown to increase the overall lifetime risk of breast cancer development (Boyd et al., 1992; Razzaghi et al., 2012).

In the tumor microenvironment, collagen crosslinking enhances integrin activation, focal adhesion maturation, PI3K/AKT signaling and tumor cell invasion/metastasis (Levental et al., 2009; Pickup et al., 2013; Rubashkin et al., 2014). High LOX expression in mammary tumors drives ECM stiffening, focal adhesion formation and metastasis, which can be abrogated using a LOX inhibitor (Levental et al., 2009; Pickup et al., 2013). Mechanistically, focal adhesion stabilization by vinculin in response to rigid ECM stimulates the activation of PI3K/AKT signaling and promotes tumor cell proliferation and invasion (Levental et al., 2009; Pickup et al., 2013; Rubashkin et al., 2014). Substrate rigidity-induced actomyosin contractility, also increases invadopodia formation and matrix degradation (Aung et al., 2014; Jerrell and Parekh, 2014), cellular responses associated with tumor aggression. In addition to a rigid matrix, integrin clustering can be facilitated by a bulky glycocalyx at the cell surface, which applies tension to matrix-bound integrins (Paszek et al., 2014). Hence, ECM stiffening through collagen crosslinking stimulates tumor cells to generate higher intracellular tension and exert stronger traction forces on their surroundings, which subsequently increases ECM stiffness as a result of applied tension. This positive feedback promotes tumor progression and metastasis.

Tumor progression propagated by ECM rigidity involves altered micro-RNA (miR) expression. Upregulation of oncogenic miR-18a in response to integrin signaling was shown to decrease PTEN expression and promote malignancy, in breast cancer models (Mouw et al., 2014). Similarly, increased ECM stiffness downregulates tumor suppressive miRs, such as miR-203. Downregulation of miR-203 was shown to repress ROBO1, which regulates actin organization and epithelial contraction (Le et al., 2016).

At the tumor periphery, increased ECM tension promotes remodeling that favors linear reorientation of collagen fibers. This ECM organization, along with compression-induced solid stress and a larger interstitial fluid pressure gradient (resulting in increased fluid flow), may promote tumor cell escape from the primary tumor. While cell migration through the ECM is impeded by protein density (small pore size) (Wirtz et al., 2011), metastasis is facilitated by oriented collagen fibers and paracrine signals from immune cells that guide the directional migration of tumor cells toward the vasculature (Condeelis and Pollard, 2006; Leung et al., 2017). Accordingly, ECM stiffening and tumor grade are associated with increase immune cell infiltration (Acerbi et al., 2015).

Remodeling of the ECM can cause the release and activation of growth factors (e.g., TGF-beta) in the stroma which further potentiate tumor progression. Matrix stiffness and TGF-beta can both drive EMT and promote tumor metastasis by increasing MMP secretion and cell migration (Wei et al., 2015). Likewise, increased ECM stiffness and TGF-beta release also cause fibroblast-to-myofibroblast conversion. Tumor associated myofibroblasts display stronger actomyosin contractility than their resident fibroblast precursors and exacerbate the desmoplastic stromal response by secreting and remodeling ECM proteins (Kumar and Weaver, 2009). Infiltrating immune cells also contribute to the desmoplastic response by secreting a variety of cytokines that activate the surrounding tumor and stromal cells. Intriguingly, tumor-activated macrophages are more compliant than resting macrophages (Yu et al., 2011).

Tumor-associated vasculature displays heightened actomyosin contractility (Yu et al., 2011) and endothelial cells plated on different ECM substrates have different responses to tension (Collins et al., 2014). As increased cell-ECM tension can disrupt cell–cell adhesions, augmented ECM stiffness and changes to the ECM composition may promote hyper-permeability of the already leaky blood vasculature and further impair tissue drainage by the lymphatic vasculature.

## Potential therapeutic targeting

In order to save lives, novel approaches to cancer prevention and treatment are needed. Given the wealth of evidence supporting physical forces as drivers of tumor growth and metastasis, and the differences in the mechanical properties of cells and matrix components between normal tissue and cancer tissue, it is reasonable to target the mechanical features of tumors for therapeutic intervention. This could be achieved through modulation of the mechanical properties of the stroma or inhibition of the cellular responses to increased stromal stiffening. Discussed next are prospective cancer therapeutics that target ECM stiffness, cell contractility or solid stress.

### Inhibition of cell contractility

Targeting cell tension, by using drugs that inhibit signaling downstream of focal adhesions to reduce actomyosin contractility, has shown promise in blocking tumor progression. In genetic mouse models of pancreatic cancer, treatment with ruxolitinib, an inhibitor of JAK, significantly reduced STAT3 activity, collagen fibrillogenesis, and ECM stiffening (Laklai et al., 2016). While ruxolitinib has been FDA approved for the treatment of myeloproliferative neoplasms (reviewed in Santos and Verstovsek, 2012), a phase 2 clinical trial of ruxolitinib demonstrated little efficacy in the treatment of human pancreatic tumors (Hurwitz et al., 2015). Several clinical trials of ruxolitinib in breast cancer and leukemia are currently under way (https://clinicaltrials.gov/).

Treatment of mice bearing patient-derived pancreatic tumors with a ROCK inhibitor (Fasudil) enhanced the effectiveness of standard chemotherapies (Gemcitabine/Abraxane) (Vennin et al., 2017). Pre-clinical studies with a FAK inhibitor (VS-4718) in a PDAC mouse model demonstrated diminished tumor fibrosis, inhibited tumor progression, increased effectiveness of chemotherapy (Gemcitabine), and an augmented responsiveness to immunotherapy (αPD1) attributed to higher levels of infiltrating CD8^+^ cytotoxic T lymphocytes (Jiang et al., 2016). These studies show the promise of enhanced therapeutic efficacy when combining drugs that target cell contractility with chemotherapy and immunotherapies. FAK inhibition is also being tested in clinical trials as a therapeutic strategy to inhibit cell contractility in various types of solid tumors (reviewed in Golubovskaya, 2014). To date, ten clinical trials aimed at testing various FAK inhibitors (PF-00562271, Defactinib/VS-6063/PF-04554878, VS-4718, GSK-2256098) in cancer patients have been completed; however, the study results are pending for all but the first phase 1 trial which assessed the safety and tolerability of PF-00562271 (https://clinicaltrials.gov/ct2/show/NCT00666926) (Infante et al., 2012).

Interestingly, retinoic acid treatment of pancreatic (PDAC) tumors also decrease cell tension in response to strain (Chronopoulos et al., 2016), suggesting that this pathway may be of therapeutic utility. Alternatively, molecular mediators of invadopodia formation could also be targeted in order to reduce metastatic spread. Furthermore, given their newfound involvement in cancer, mechanically-activated ion channels (such as Piezo1) are also potential therapeutic targets. However, some caution is needed as some side effects of the chemotherapeutic drug cisplatin, have been attributed to its non-specific inhibition of mechanosensitive ion channels (Milosavljevic et al., 2010).

### Targeting solid stress and interstitial fluid pressure

Solid stress compresses the vasculature and leads to hypoxia, impaired drug delivery, and higher interstitial fluid pressure. Thus, approaches have been developed to target solid and fluid stresses within the tumor microenvironment. Examples include hyaluronidases and angiotensin inhibitors. Hyaluronidases (e.g., PEGPH20), degrade the ECM protein hyaluronan to release immobilized fluid and improve tissue compliance (Whatcott et al., 2011). Angiotensin inhibitors (e.g., losartan) cause dilation of the vasculature and blood pressure reduction, thereby decreasing interstitial fluid pressure and enabling better perfusion and therapeutic efficacy. In breast and pancreatic cancers, inhibiting angiotensin with losartan decompressed tumor blood vessels and reduced stromal collagen, which increased vascular perfusion and improved drug delivery (Chauhan et al., 2013). Furthermore, losartan was shown to inhibit mammary tumor development and progression, as well as increase in blood vessel diameter, by inhibiting AT1R activation (Coulson et al., 2017). Both PEGPH20 and losartan are currently in clinical trials for the treatment of solid tumors (https://clinicaltrials.gov/) (results of completed studies were not available at time of publication).

### Modulation of ECM stiffness

The desmoplastic stromal response is common to several types of solid tumors, including pancreatic and breast cancers, and is a major impediment to treatment as it increases tissue stresses and impairs drug delivery. Increased collagen deposition by stromal fibroblasts largely contributes to the fibrosis in the tumor microenvironment. Several drugs have emerged as potent stromal inhibitors. In a mouse model of breast cancer, targeting COX-2 with celecoxib significantly reduced αSMA-positive cancer-associated fibroblasts, inhibited immune cell recruitment and decreased collagen deposition, tumor growth and metastasis (Esbona et al., 2016). Celecoxib (Celebrex), which is clinically approved to treat inflammatory diseases, has been clinically demonstrated to improve cancer treatment outcomes but can cause serious adverse cardiovascular events, potentially reducing its utility as an anti-cancer agent (reviewed in Chen et al., 2014). Inhibitors of TGF-beta and hedgehog signaling (in combination with standard chemotherapy) are also attractive therapeutic targets to prevent desmoplasia, solid stress and tumor progression (Ko et al., 2016, reviewed in Neuzillet et al., 2015).

Disruption of ECM stiffening using LOX inhibition, (e.g., BAPN) is a potential therapeutic strategy that has been shown to greatly reduce tumor aggression in pre-clinical models of breast cancer (Levental et al., 2009; Pickup et al., 2013; Mouw et al., 2014). Another approach to targeting ECM remodeling is inhibition of matrix metalloproteinase; however, these inhibitors have shown little clinical promise (reviewed in Coussens et al., 2002). Lastly, given the recent success of immunotherapies in the treatment of cancer and the association between ECM stiffness, immune cell infiltration and tumor grade (Acerbi et al., 2015), it is conceivable that drugs which inhibit immune cell infiltration or modulate immune cell responses (e.g., simvastatin, metformin) may also improve cancer outcomes (Incio et al., 2015).

A potential limitation to all of these approaches is the high variability in tumor phenotype between tumors, and even within the same tumor, which could reduce therapeutic effectiveness. This is exemplified by heterogeneity in cellular response to stiffness between GBM tumors of same type (Grundy et al., 2016), likely owing to underlying genetic variability. Nevertheless, these approaches hold much promise for the treatment of cancers and the most effective therapeutic strategies will probably impact multiple sources of stress within the tumor microenvironment.

## Summary

**Figure 4:**
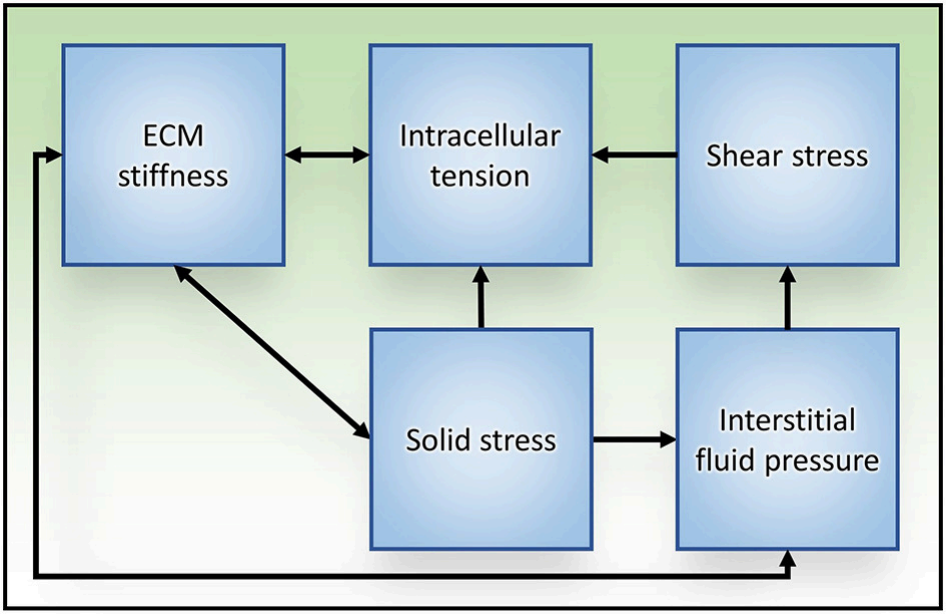
Relationships between mechanical stress and tissue responses during tumor progression to metastasis. Increased intracellular tension is produced through actomyosin contraction in response to both biochemical and mechanical stimuli. ECM stiffening stimulates cells to generate higher intracellular tension to exert stronger traction forces on their surroundings, which subsequently exacerbates ECM stiffness. Solid stress is caused in part by unchecked proliferation of cancer cells that results in expansion of the tumor mass, compression of the tumor interior and distention of the surrounding stromal tissue. ECM stiffness can increase solid stress by augmenting the resistance to tumor expansion. Reciprocally, tumor expansion causes circumferential ECM tension and tissue stiffening. As a result of tissue compression and ECM stiffening, blood and lymphatic vascular function is impaired (due to vascular crushing) and interstitial fluid pressure is increased. Aberrant fluid flow throughout the interstitial spaces and within the obstructed tumor vasculature increases the shear stress experienced by tumor cells.

Within the tumor microenvironment, tumor cells are exposed to a number of different mechanical stimuli including cell–cell and cell-ECM tension, compression stress from the expanding tumor mass, interstitial fluid pressure, and shear stress. Furthermore, positive feedback exists between these stimuli and the responses they elicit (Figure 4), such as increased cellular actomyosin contractility and ECM stiffening, which exacerbates tumor progression and aggression. While this review focused on solid tumors, these physical interactions are universally applicable to all types of malignancies. Thus, understanding the physical interactions between components of the tumor microenvironment and the molecular mechanisms regulating cellular responses to mechanical inputs will be key to developing effective therapeutics to treat all cancers.

## Author contributions

JN and ID: drafted the article. JN, JM, and VW: revised and edited the article.

### Conflict of interest statement

The authors declare that the research was conducted in the absence of any commercial or financial relationships that could be construed as a potential conflict of interest.
